# Effect of Amblyopia Therapy on Retinal and Choroidal Thickness in Children With Unilateral Anisometropic Amblyopia: A Prospective Longitudinal Study

**DOI:** 10.7759/cureus.107910

**Published:** 2026-04-28

**Authors:** Shreya Bhadani, Pallavi P Sahu, Shovna Dash, Soumyakanta Mohanty, Aanchal Singhal, Manisha Choudhary

**Affiliations:** 1 Ophthalmology, Kalinga Institute of Medical Sciences, Bhubaneswar, IND

**Keywords:** anisometropic amblyopia, foveal thickness, macular thickness, oct (optical coherence tomography), retinal nerve fiber layer thickness, subfoveal choroidal thickness

## Abstract

Introduction

Anisometropic amblyopia is a leading cause of preventable monocular visual impairment in children. Traditionally considered a cortical developmental disorder, recent advances in optical coherence tomography (OCT) have demonstrated measurable structural alterations in the retina and choroid of amblyopic eyes. These findings suggest that amblyopia may also involve retinal and choroidal components.

Objective

To evaluate longitudinal changes in retinal and choroidal thickness following amblyopia therapy in children with unilateral anisometropic amblyopia.

Methods

This prospective longitudinal study included 26 children aged 6-18 years diagnosed with unilateral anisometropic amblyopia. Best-corrected visual acuity (BCVA), foveal thickness (FT), macular thickness (MT), subfoveal choroidal thickness (SFCT), and retinal nerve fiber layer thickness (RNFLT) were assessed in both amblyopic and fellow eyes using OCT. Measurements were obtained at baseline and at 3, 6, and 12 months following amblyopia therapy. Baseline interocular comparisons and longitudinal changes in the amblyopic eye were analyzed.

Results

At baseline, the mean BCVA of the amblyopic eye was 0.70 ± 0.18 logMAR compared to 0.00 logMAR in the fellow eye. Mean baseline FT was 267.63 ± 15.1 µm in amblyopic eyes versus 233.83 ± 11.4 µm in fellow eyes. Mean MT was 278.90 ± 16.8 µm versus 244.10 ± 12.6 µm, SFCT was 355.31 ± 27.1 µm versus 280.63 ± 18.9 µm, and RNFLT was 103.85 ± 6.9 µm versus 98.66 ± 5.3 µm in amblyopic and fellow eyes, respectively. During follow-up, mean BCVA improved progressively from 0.70 ± 0.18 logMAR at baseline to 0.40 ± 0.15 at 3 months, 0.30 ± 0.13 at 6 months, and 0.20 ± 0.10 at 12 months. Mean FT decreased from 267.63 ± 15.1 µm to 252.10 ± 14.3 µm, MT from 278.90 ± 16.8 µm to 263.20 ± 16.2 µm, and SFCT from 355.31 ± 27.1 µm to 334.60 ± 25.7 µm over 12 months. RNFLT remained relatively unchanged throughout the study period.

Conclusions

Children with unilateral anisometropic amblyopia show increased retinal and choroidal thickness compared to the fellow eye. With treatment, visual acuity improves significantly, along with reductions in foveal, macular, and SFCT, while RNFLT remains largely unchanged. However, these structural changes should be interpreted as associations rather than definite effects of therapy, given the presence of potential confounding factors.

## Introduction

Amblyopia is one of the most common causes of preventable monocular visual impairment in children and young adults [1,2]. It is defined as reduced best-corrected visual acuity (BCVA) that cannot be explained by structural ocular abnormality alone and results from abnormal visual experience during the critical period of visual development. Among its subtypes, anisometropic amblyopia is particularly important because unequal refractive errors between the two eyes lead to chronic blur in one eye, suppression, and abnormal cortical maturation [3].

Traditionally, amblyopia has been regarded primarily as a cortical disorder. However, with advances in optical coherence tomography (OCT) and enhanced depth imaging (EDI), increasing attention has been directed toward possible structural alterations in the retina and choroid of amblyopic eyes [4,5,6]. Previous studies demonstrate that children with unilateral anisometropic amblyopia exhibit increased retinal and choroidal thickness in the amblyopic eye compared with the fellow eye at baseline [7,8].

This study aimed to evaluate changes in macular thickness (MT), foveal thickness (FT), retinal nerve fiber layer thickness (RNFLT), and subfoveal choroidal thickness (SFCT) following amblyopia therapy [9,10]. Additionally, the study sought to assess longitudinal changes in these parameters and compare them with those of the fellow eye at baseline in children with unilateral anisometropic amblyopia.

## Materials and methods

This prospective longitudinal study was conducted in the Department of Ophthalmology at Kalinga Institute of Medical Sciences, Bhubaneswar, Odisha, India. Children aged 6-18 years with unilateral anisometropic amblyopia were included. OCT-derived retinal and choroidal parameters were evaluated before the initiation of therapy and during follow-up.

Study period

The study was conducted over a period of two years, from February 2024 to February 2026.

Inclusion and exclusion criteria 

Children aged 6-18 years of either sex with unilateral anisometropic amblyopia, defined as an interocular difference in BCVA of ≥0.2 logMAR, were included in the study.

Patients were excluded if they had a prior history of occlusion therapy or ocular surgery; presence of strabismus, nystagmus, hearing impairment, or developmental delay; or were non-compliant with spectacle wear or demonstrated poor fixation and cooperation during spectral-domain optical coherence tomography (SD-OCT) assessment.

Sample size calculation

The sample size was estimated using previously published data by Heralgi et al. (2019), based on the mean and standard deviation of MT at 12 months in amblyopic and normal eyes (248.90 ± 11.681 µm and 239.47 ± 5.569 µm, respectively). As suitable paired longitudinal OCT data were not available at the time of study planning to directly estimate within-subject variability or interocular correlation, these intergroup data were used as a pragmatic approximation to determine the expected effect size.

We acknowledge that this approach does not account for within-subject variability and interocular correlation inherent to the paired longitudinal study design. However, in the present study, the fellow eye was used as an internal control, and statistical analysis was performed using paired and repeated-measures methods to appropriately account for interocular comparisons and longitudinal changes.

The sample size for comparing two means was calculated using the following formula [11]:

 n = (Z_α/2_​+Z_β_​)^2^(σ^2^_1_​+σ^2^_2_​)​/(μ_1_​−μ_2​_)^2^

where n = sample size per group

Z_α/2_​ = 1.96 for 5% level of significance

Z_β_​ = 1.645 for 95% power σ_1​_,σ_2_​ = standard deviations of the two groups

μ_1_​-μ_2_​ = expected mean difference

Sample size 

The calculated sample size was approximately 25 eyes per group. To account for rounding and feasibility, 26 participants were included in the study. The fellow eye was used as an internal control for comparison.

Data analysis

Data were entered into Microsoft Excel and analyzed using IBM SPSS Statistics version 25.0 (IBM Corp., Armonk, NY, USA). Continuous variables were expressed as mean ± standard deviation.

Interocular comparisons between amblyopic and fellow eyes were performed using the paired t-test. Longitudinal changes across follow-up visits were analyzed using repeated-measures ANOVA, followed by post hoc analysis where appropriate.

A p-value of <0.05 was considered statistically significant.

Study procedure

For this study, the following parameters were analyzed: BCVA, FT, MT, SFCT, and RNFLT. Measurements were recorded at four visits: day 0, 3 months, 6 months, and 12 months. Continuous variables are presented as mean ± standard deviation.

OCT imaging was performed using spectral-domain OCT (Heidelberg Spectralis). Enhanced depth imaging (EDI) mode was used for choroidal assessment. Macular scans were obtained using a standardized protocol with automated segmentation, and all scans were reviewed for segmentation accuracy. Scan averaging was performed, for example, 9-25 frames, to improve image quality. Only scans with adequate image quality (Q ≥ 20-25), stable fixation, and accurate segmentation were included in the analysis. Standardized acquisition protocols, including eye tracking (TruTrack), scan averaging, and consistent imaging conditions, were used. All measurements were obtained by a single experienced operator to minimize variability.

All patients were initially prescribed full-time spectacle correction based on cycloplegic refraction, followed by a refractive adaptation period of approximately 4-6 weeks. Subsequently, occlusion therapy was initiated and tailored according to the severity of amblyopia, typically ranging from 2 to 6 hours per day. Active vision therapy, incorporating perceptual learning techniques, anaglyph glasses, and structured video game-based interventions, was also administered and was found to be effective in the treatment of anisometropic amblyopia as well as in improving visual acuity. The treatment regimen was individualized based on clinical judgment and patient response. Compliance was assessed at each follow-up visit through parental diaries and caregiver interviews, and variations in adherence were documented and considered during analysis.

Ethical approval

This study was approved by the Institutional Ethics Committee of Kalinga Institute of Medical Sciences, Bhubaneswar, Odisha, India (Approval No.: KIIT/KIMS/IEC/1521/2024). The study adhered to the tenets of the Declaration of Helsinki. Written informed consent was obtained from the parents or legal guardians of all participants, and assent was obtained from children where applicable.

## Results

Table 1 illustrates a baseline comparison of functional and retinal-choroidal parameters between amblyopic and fellow eyes in children with unilateral anisometropic amblyopia. The amblyopic eye demonstrates significantly reduced visual acuity, with a mean BCVA of 0.70 ± 0.18 logMAR compared to 0.00 logMAR in the fellow eye, confirming the presence of marked visual impairment.

**Table 1:** Baseline comparison of visual acuity and retinal-choroidal thickness parameters between amblyopic and fellow eyes. BCVA: Best-corrected visual acuity; logMAR: Logarithm of the minimum angle of resolution; FT: Foveal thickness; MT: Macular thickness; SFCT: Subfoveal choroidal thickness; RNFLT: Retinal nerve fiber layer thickness.

Parameter	Amblyopic eye (mean ± SD)	Fellow eye (mean ± SD)
BCVA (logMAR)	0.70 ± 0.18	0
FT (µm)	267.63 ± 15.1	233.83 ± 11.4
MT (µm)	278.90 ± 16.8	244.10 ± 12.6
SFCT (µm)	355.31 ± 27.1	280.63 ± 18.9
RNFLT (µm)	103.85 ± 6.9	98.66 ± 5.3

In addition to the functional deficit, structural differences appear to be evident. FT and MT are higher in the amblyopic eye (267.63 ± 15.1 µm and 278.90 ± 16.8 µm, respectively) compared to the fellow eye (233.83 ± 11.4 µm and 244.10 ± 12.6 µm), indicating relatively increased retinal thickness. Similarly, SFCT is greater in the amblyopic eye (355.31 ± 27.1 µm) than in the fellow eye (280.63 ± 18.9 µm), suggesting possible choroidal involvement in amblyopia.

RNFLT is also slightly increased in the amblyopic eye (103.85 ± 6.9 µm vs. 98.66 ± 5.3 µm), although this difference is less pronounced compared to other parameters. Overall, the table suggests that amblyopia may be associated not only with reduced visual acuity but also with measurable structural differences in both retinal and choroidal layers, which may support the concept that amblyopia involves anatomical as well as functional components.

Table 2 illustrates the longitudinal improvement in BCVA in the amblyopic eye over a 12-month period following treatment. At baseline (day 0), the mean BCVA was 0.70 ± 0.18 logMAR, indicating significant visual impairment. Following the initiation of therapy, there is a marked improvement by month 3, with BCVA improving to 0.40 ± 0.15 logMAR. This early improvement suggests a strong initial response to treatment, likely due to optical correction and the onset of occlusion therapy.

**Table 2:** Longitudinal changes in best-corrected visual acuity (BCVA) in the amblyopic eye over 12 months. BCVA: Best-corrected visual acuity; logMAR: Logarithm of the minimum angle of resolution.

Visit	Mean BCVA (logMAR) ± SD	p-value
Day 0	0.70 ± 0.18	-
Month 3	0.40 ± 0.15	<0.001
Month 6	0.30 ± 0.13	<0.001
Month 12	0.20 ± 0.10	<0.001

Further gradual improvement is observed at month 6 (0.30 ± 0.13 logMAR) and continues up to month 12 (0.20 ± 0.10 logMAR), indicating sustained therapeutic benefit over time. The rate of improvement appears to be more pronounced in the initial months, followed by slower, steady progression, which is characteristic of the amblyopia treatment response. The changes from baseline at all follow-up visits are statistically significant (p < 0.001), confirming that the observed improvements are unlikely to be due to chance.

Overall, this table demonstrates that amblyopia therapy leads to consistent and clinically meaningful enhancement in visual acuity, highlighting the effectiveness of early and sustained intervention in children with anisometropic amblyopia.

**Figure 1:**
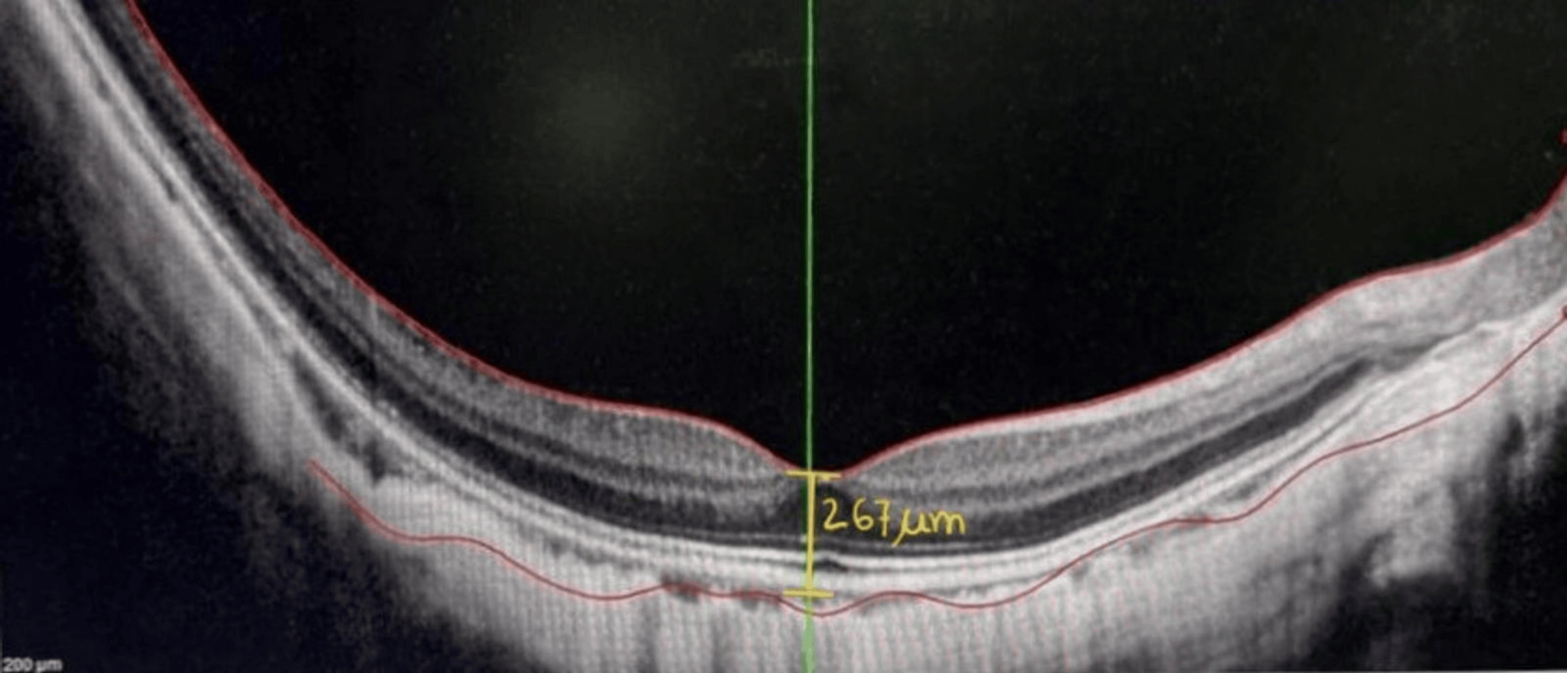
Macular OCT demonstrating increased central macular thickness before therapy. OCT: Optical coherence tomography.

**Figure 2:**
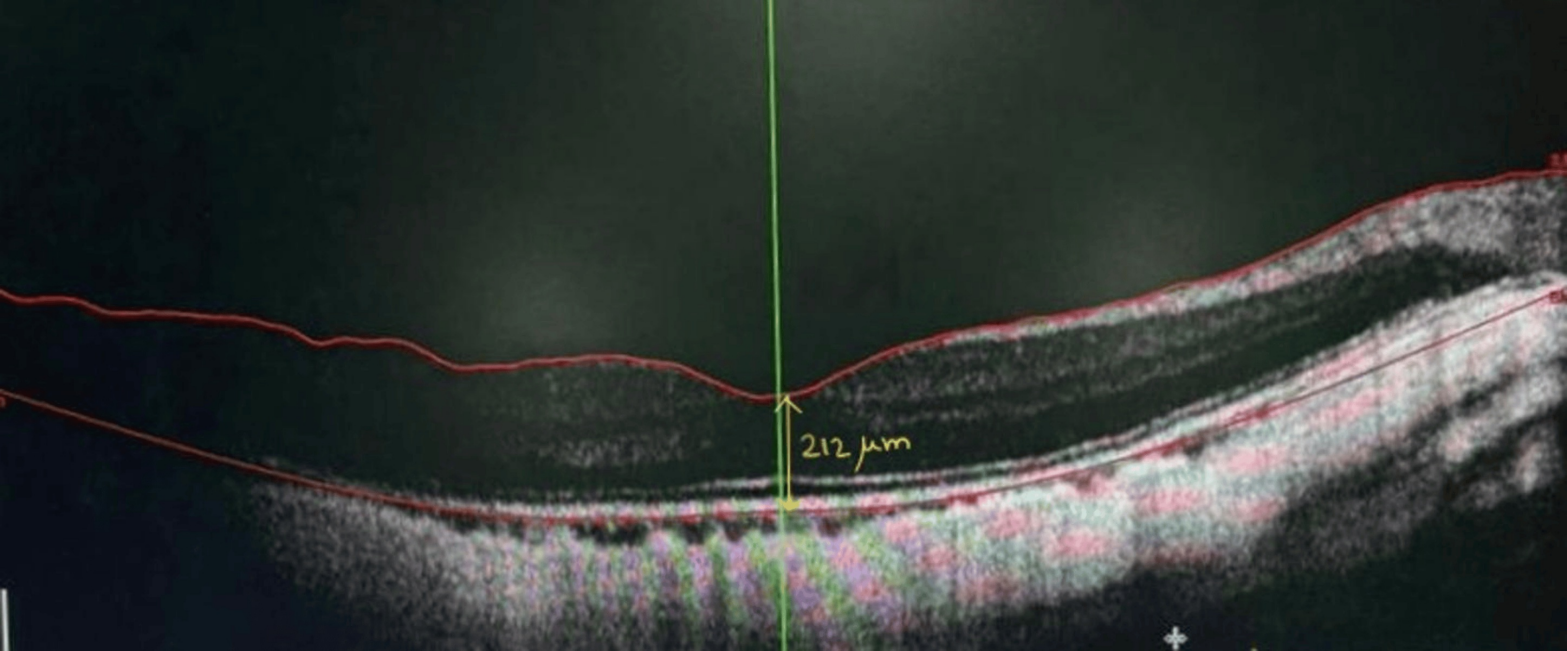
Macular OCT showing reduced central macular thickness after therapy. OCT: Optical coherence tomography.

Table 3 demonstrates the longitudinal changes in retinal and choroidal thickness parameters in the amblyopic eye over a 12-month follow-up period. A gradual reduction is observed in FT, MT, and SFCT following amblyopia therapy. FT decreases from 267.63 ± 15.1 µm at baseline to 252.10 ± 14.3 µm at 12 months, while MT decreases from 278.90 ± 16.8 µm to 263.20 ± 16.2 µm over the same period. Representative macular OCT images demonstrate increased central macular thickness before therapy and a reduction after therapy (Figures 1-2).

**Table 3:** Longitudinal changes in retinal and choroidal thickness in the amblyopic eye over 12 months. FT: Foveal thickness; MT: Macular thickness; SFCT: Subfoveal choroidal thickness; RNFLT: Retinal nerve fiber layer thickness. All measurements are reported in micrometers (µm).

Visit	FT (µm), mean ± SD	MT (µm), mean ± SD	SFCT (µm), mean ± SD	RNFLT (µm), mean ± SD
Day 0	267.63 ± 15.1	278.90 ± 16.8	355.31 ± 27.1	103.85 ± 6.9
Month 3	261.80 ± 14.6	274.70 ± 17.4	346.20 ± 26.3	103.40 ± 6.4
Month 6	258.90 ± 13.8	269.40 ± 15.9	339.80 ± 24.9	103.70 ± 6.8
Month 12	252.10 ± 14.3	263.20 ± 16.2	334.60 ± 25.7	103.30 ± 6.3
p-value	<0.001	<0.001	<0.001	>0.05

**Figure 3:**
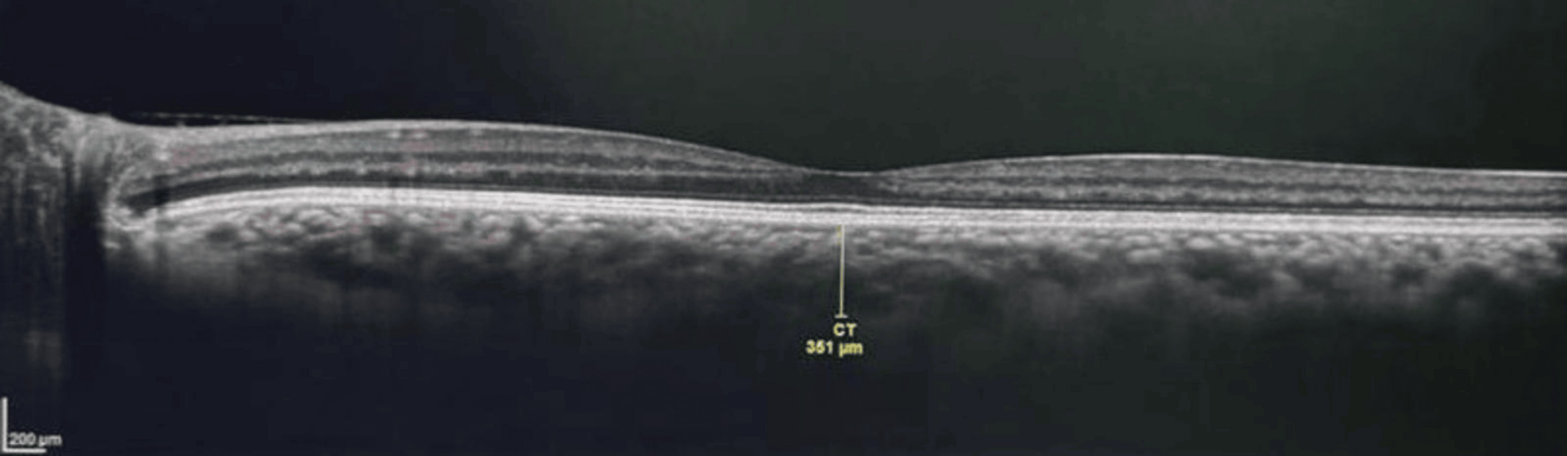
Enhanced depth OCT showing increased subfoveal choroidal thickness before therapy. OCT: Optical coherence tomography.

**Figure 4:**
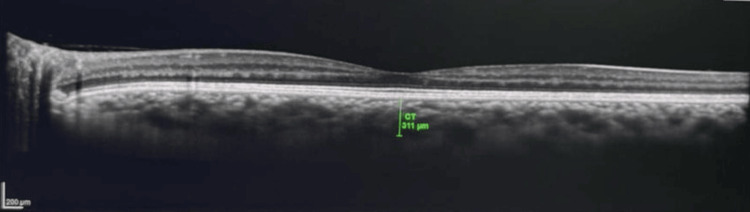
Enhanced depth OCT showing reduced subfoveal choroidal thickness after therapy. OCT: Optical coherence tomography.

Similarly, SFCT shows a decline from 355.31 ± 27.1 µm to 334.60 ± 25.7 µm, suggesting a reduction in choroidal thickness over time. Representative enhanced depth OCT images show increased subfoveal choroidal thickness before therapy and a reduction after therapy (Figures 3-4). These reductions appear progressive, with relatively more noticeable changes in the earlier follow-up visits and gradual continuation thereafter. The changes in FT, MT, and SFCT are statistically significant (p < 0.001), indicating that these structural variations are associated with the treatment period, although they should be interpreted with appropriate caution.

In contrast, RNFLT remains relatively stable throughout the study period (103.85 ± 6.9 µm at baseline and 103.30 ± 6.3 µm at 12 months), and this variation is not statistically significant (p > 0.05). This may suggest that inner retinal layers are less affected over the course of therapy. Overall, the findings may indicate that structural changes in amblyopia predominantly involve the outer retina and choroid rather than the RNFL.

Table 4 presents the longitudinal changes in functional and retinal-choroidal parameters in the fellow eye over a 12-month follow-up period. The values for BCVA remain constant at 0.00 logMAR across all visits, indicating stable visual function throughout the study period. Similarly, structural parameters, including FT, MT, SFCT, and RNFLT, show minimal to no appreciable variation over time.

**Table 4:** Longitudinal changes in functional and retinal-choroidal parameters in the fellow eye from baseline to 12 months. BCVA: Best-corrected visual acuity; logMAR: Logarithm of the minimum angle of resolution; FT: Foveal thickness; MT: Macular thickness; SFCT: Subfoveal choroidal thickness; RNFLT: Retinal nerve fiber layer thickness. All thickness measurements are reported in micrometers (µm).

Visit	BCVA (logMAR)	FT (µm), mean ± SD	MT (µm), mean ± SD	SFCT (µm), mean ± SD	RNFLT (µm), mean ± SD
Day 0	0	233.83 ± 11.4	244.10 ± 12.6	280.63 ± 18.9	98.66 ± 5.3
Month 3	0	234.20 ± 12.0	244.50 ± 13.3	281.10 ± 19.5	98.40 ± 5.1
Month 6	0	233.50 ± 11.1	243.90 ± 12.7	280.20 ± 18.2	98.90 ± 5.6
Month 12	0	233.90 ± 11.7	244.30 ± 13.0	280.80 ± 18.7	98.50 ± 5.2
p-value	>0.05	>0.05	>0.05	>0.05	>0.05

The absence of measurable change across visits is supported by non-significant p-values (p > 0.05) for all parameters. These findings suggest that the fellow eye remains structurally and functionally stable during the study period and does not appear to be influenced by amblyopia therapy administered to the amblyopic eye.

This relative stability supports the use of the fellow eye as an internal reference for comparison, while acknowledging its inherent limitations, and suggests that the observed changes in the amblyopic eye are unlikely to be explained by natural variation or measurement error alone.

Table 5 summarizes the key functional and structural changes observed in the amblyopic eye from baseline to 12 months following therapy. There is a clear improvement in visual acuity, with BCVA improving from 0.70 ± 0.18 to 0.20 ± 0.10 logMAR, which is statistically significant (p < 0.001). Structural parameters show a consistent reduction over time.

**Table 5:** Summary of key changes from baseline to 12 months in the amblyopic eye. BCVA: Best-corrected visual acuity; logMAR: Logarithm of the minimum angle of resolution; FT: Foveal thickness; MT: Macular thickness; SFCT: Subfoveal choroidal thickness; RNFLT: Retinal nerve fiber layer thickness. All thickness measurements are expressed in micrometers (µm).

Parameter	Baseline (mean ± SD)	12 months (mean ± SD)	Direction of change	p-value
BCVA (logMAR)	0.70 ± 0.18	0.20 ± 0.10	Improved	<0.001
FT (µm)	267.63 ± 15.1	252.10 ± 14.3	Decreased	<0.001
MT (µm)	278.90 ± 16.8	263.20 ± 16.2	Decreased	<0.001
SFCT (µm)	355.31 ± 27.1	334.60 ± 25.7	Decreased	<0.001
RNFLT (µm)	103.85 ± 6.9	103.30 ± 6.3	Unchanged	>0.05

FT, MT, and SFCT all demonstrate decreases, suggesting changes in retinal and choroidal structures over the treatment period. These changes are also statistically significant (p < 0.001). In contrast, RNFLT remains relatively stable throughout the study period, with no statistically significant change (p > 0.05).

These findings may suggest that amblyopia predominantly involves outer retinal and choroidal structures rather than the inner retinal layers. Overall, the table highlights both functional improvement and associated structural changes observed during amblyopia therapy, which should be interpreted within the appropriate clinical context. 

## Discussion

In this study, children with unilateral anisometropic amblyopia were found to have increased retinal and choroidal thickness in the amblyopic eye relative to the fellow eye at baseline. Specifically, FT, MT, SFCT, and RNFLT were higher in the amblyopic eye.

These findings are consistent with previous OCT-based studies by Aygit ED et al. [4] and Nishi T et al. [5], which also reported increased choroidal and MT in anisometropic amblyopia. A key observation in this study is the progressive improvement in BCVA over the 12-month follow-up period, accompanied by reductions in FT, MT, and SFCT [9,10]. Similar findings have been reported by Araki S et al. [9], who demonstrated improvement in visual acuity along with a decrease in choroidal and MT following treatment.

Among the OCT parameters, SFCT showed the most pronounced reduction over time, followed by FT and MT [5,6,12]. This may support the hypothesis that the choroid could play a role in ocular growth regulation and the pathophysiology of anisometropic amblyopia. In contrast, RNFLT remained relatively unchanged throughout the follow-up period [13-15]. This finding may suggest that the inner retinal layers are less affected by amblyopia or potentially less responsive to therapy. Similar observations have been reported by Dickmann A et al. [13] and Kee SY et al. [14], who found no significant change in RNFL thickness in amblyopic eyes, although results across studies remain inconsistent. The improvement in BCVA observed in this study is in agreement with established amblyopia treatment outcomes and dose-response relationships described in earlier studies [16,17,18].

The observed changes in retinal and choroidal thickness should be interpreted with caution. Due to potential confounding factors and variability in treatment, these findings are better considered associations rather than definitive effects of therapy. Similar OCT-based studies report structural differences without establishing causality, and the changes observed may reflect adaptive variations rather than direct treatment effects.

The strengths of this study include its prospective longitudinal design and the use of the fellow eye as an internal control, which helps minimize intersubject variability. Additionally, the study focuses specifically on a pediatric population with unilateral anisometropic amblyopia, allowing for a more controlled evaluation of structural changes observed over the study period.

The relatively lower variability in this study may be explained by the use of standardized imaging protocols, such as eye tracking, scan averaging, and single-operator measurements under consistent conditions, which help improve repeatability and reduce measurement variability in OCT imaging.

Formal repeatability analysis, such as intra-observer variability assessment or intraclass correlation coefficient calculation, was not performed, which may limit the ability to fully assess measurement consistency.

The sample size estimation was based on intergroup data and did not account for within-subject correlation inherent to the paired study design, which represents a methodological limitation.

The present study is limited by a relatively small sample size, which may affect the generalizability of the results. The lack of an independent control group, with reliance on the fellow eye, may introduce bias, as the fellow eye may not fully represent a truly normal population, particularly in anisometropic amblyopia, where bilateral influences may exist. However, its use allows for within-subject comparison and helps reduce interindividual variability. Additionally, variations in patient compliance may have influenced the outcomes. These factors represent important limitations and should be carefully considered when interpreting the findings.

We recognize that factors such as axial length, refractive error, and diurnal variation can influence OCT measurements, particularly choroidal thickness. These were not specifically controlled or adjusted for in the present study and may have had some effect on the results. However, using the fellow eye as an internal control allowed for within-subject comparison, which helps reduce differences between individuals. In addition, all OCT scans were performed under consistent conditions, including similar timing, standardized protocols, and measurements by a single experienced operator, which may improve reliability. Nonetheless, the absence of direct control for these factors remains a limitation and should be kept in mind when interpreting the findings.

The treatment protocol was not standardized, as patients received individualized combinations of refractive correction, occlusion therapy, and vision therapy. Therefore, the observed structural changes reflect the overall effect of amblyopia management rather than the impact of any specific treatment modality. This approach was intended to mirror real-world clinical practice, where treatment is tailored according to patient age, severity of amblyopia, and compliance. Consequently, while the findings demonstrate structural and functional improvement over time, they do not allow definitive attribution of these changes to a single therapeutic intervention.

We used repeated-measures ANOVA for longitudinal and within-subject comparisons, which is an accepted approach. However, it may not fully account for all confounders or intra-subject variability. More advanced methods, such as mixed-effects models, could provide a more robust analysis. Given the sample size and study objectives, the chosen method was considered appropriate, but this remains a limitation and should be considered when interpreting the findings. Future studies may incorporate these models for better adjustment.

## Conclusions

Children with unilateral anisometropic amblyopia tend to show increased retinal and choroidal thickness in the amblyopic eye at baseline. With treatment, visual acuity improves significantly, along with reductions in foveal thickness, macular thickness, and subfoveal choroidal thickness, while RNFLT remains largely unchanged. These findings suggest that amblyopia may involve both functional and structural changes; however, the observed structural changes should be interpreted as associations rather than definite effects of therapy, considering the presence of potential confounding factors. OCT remains a useful, non-invasive tool for monitoring these changes over time.
